# Low-Density Lipoprotein Cholesterol Reduction and Therapeutic Adherence During Cardiac Rehabilitation After Myocardial Infarction

**DOI:** 10.3390/jcm14124242

**Published:** 2025-06-14

**Authors:** Carlos Bertolín-Boronat, Héctor Merenciano-González, Víctor Marcos-Garcés, María Luz Martínez Mas, Josefa Inés Climent Alberola, José Manuel Civera, María Valls Reig, Marta Ruiz Hueso, Patricia Castro Carmona, Nerea Perez, Laura López-Bueno, Beatriz Díaz Díaz, Isabel Miñano Martínez, Alfonso Payá Rubio, César Ríos-Navarro, Elena de Dios, Jose Gavara, Manuel F. Jiménez-Navarro, Juan Sanchis, Vicente Bodi

**Affiliations:** 1 Department of Cardiology, Hospital Clinico Universitario de Valencia, 46010 Valencia, Spainhectormeren@gmail.com (H.M.-G.); mluzmmas@comv.es (M.L.M.M.); josemanuelciveragomez@gmail.com (J.M.C.); mvallsr@gmail.com (M.V.R.); ruiz_marhue1@gva.es (M.R.H.); castro_pat@gva.es (P.C.C.); sanchis_juafor@gva.es (J.S.); vicente.bodi@uv.es (V.B.); 2 INCLIVA Health Research Institute, 46010 Valencia, Spain; neere_8@hotmail.com (N.P.); cesar_rios1@hotmail.com (C.R.-N.); 3 Network Biomedical Research Center for Cardiovascular Diseases (CIBER-CV), 28029 Madrid, Spain; elenaddll@gmail.com (E.d.D.); jose_4_6_90@hotmail.com (J.G.);; 4 Department of Rehabilitation, Hospital Clinico Universitario de Valencia, 46010 Valencia, Spain; inescliment093@gmail.com (J.I.C.A.); laura.lopez@uv.es (L.L.-B.); beatriz.diaz@uv.es (B.D.D.); isabelmimar@gmail.com (I.M.M.); paya_alf@gva.es (A.P.R.); 5 Centre for Biomaterials and Tissue Engineering, Universitat Politènica de València, 46022 Valencia, Spain; 6 Servicio de Cardiología y Cirugía Cardiovascular-Área del Corazón, Hospital Universitario Virgen de la Victoria, 29010 Málaga, Spain; 7 Instituto de Investigación Biomédica de Málaga y Plataforma en Nanomedicina (IBIMA Plataforma BIONAND), 29590 Málaga, Spain; 8 Departamento de Medicina y Dermatología, Facultad de Medicina, Universidad de Málaga, 29010 Málaga, Spain; 9 Department of Medicine, Faculty of Medicine and Odontology, University of Valencia, 46010 Valencia, Spain

**Keywords:** residual difference, LDL cholesterol, therapeutic adherence, myocardial infarction, cardiac rehabilitation, lifestyle recommendations, Mediterranean diet, exercise testing

## Abstract

**Background:** A significant proportion of post-myocardial infarction (MI) patients do not reach target low-density lipoprotein cholesterol (LDL-C) levels. Suboptimal LDL-C reduction is often attributed to poor adherence to pharmacological therapy and lifestyle recommendations. **Methods**: In a prospective registry of 179 post-MI patients who completed a Phase 2 Cardiac Rehabilitation Program (CRP), we evaluated the characteristics and predictors of suboptimal LDL-C reduction. Key indicators were assessed before and after CRP: adherence to the Mediterranean diet (using the PREDIMED questionnaire), weekly physical activity (via the IPAQ questionnaire), therapeutic adherence (using the Morisky–Green questionnaire), and peak oxygen consumption (VO_2_) on exercise testing. Lipid-lowering therapy (LLT) and LDL-C were recorded prior to MI and both before and after Phase 2 CRP. At the end of Phase 2, we analyzed the difference between measured and theoretical LDL-C (basal LDL-C minus expected LDL-C reduction by LLT), which was defined as “residual difference in LDL-C” (RD-LDL-C). We analyzed the predictors of positive RD-LDL-C (lower than theoretically expected). **Results**: After CRP, 54 (30.2%) patients exhibited positive RD-LDL-C. Within this subgroup, LLT was uptitrated, and patients received more potent LLT at the conclusion of CRP (theoretical potency: 69.81 ± 7.07 vs. 66.41 ± 7.48%, *p* = 0.005). However, they were less likely to reach the target LDL-C level <55 mg/dL (66.7% vs. 93.6%, *p* < 0.001). Male sex (HR 17.96 [2.15, 149.92], *p* = 0.008) and higher lipoprotein (a) levels (HR 1.02 [1.01, 1.03] per mg/dL, *p* = 0.001) were associated with a positive RD-LDL-C. Conversely, diabetes mellitus (HR 0.17 [0.06, 0.51], *p* = 0.002), higher corrected basal LDL-C levels (HR 0.98 [0.97, 0.99] per mg/dL, *p* = 0.001), and supervised in-hospital training during CRP (HR 0.28 [0.09, 0.86], *p* = 0.03) were associated with a reduced probability of positive RD-LDL-C. No association was found with adherence to the Mediterranean diet (88.1%), therapeutic adherence (89.1%), reported weekly physical activity (median 3545 [1980, 6132] metabolic equivalents per week), or change in peak VO_2_. **Conclusions**: More than one-third of post-MI patients demonstrated lower than expected LDL-C reduction (positive RD-LDL-C) following CRP, a finding that could not be attributed to poor adherence to pharmacological therapy or lifestyle recommendations. These findings suggest that a personalized approach to prescribing and uptitrating LLT may help achieve LDL-C targets, particularly in MI patients with healthy lifestyle habits who exhibit a lower response to LLT.

## 1. Introduction

Extensive genetic, observational, and interventional studies have provided robust evidence that low-density lipoprotein cholesterol (LDL-C) and other apolipoprotein-B–containing lipoproteins play a fundamental role in the onset and progression of atherosclerotic cardiovascular disease [1,2]. Atherosclerosis, a condition characterized by the accumulation of cholesterol plaques within the arterial walls, is the primary underlying mechanism of cardiovascular disease, including myocardial infarction and stroke [3].

Accordingly, leading scientific societies recommend lipid profile modification, primarily through LDL-C reduction, as one of the most important secondary prevention goals following myocardial infarction (MI) [4,5,6]. In this context, Cardiac Rehabilitation Programs (CRP) after MI offer an ideal framework to address lifestyle habits, bolster secondary preventive therapy, and improve therapeutic adherence, thereby ensuring the attainment of treatment goals [7,8,9]. Furthermore, participation in a CRP has been associated with reduced hospitalization for cardiac causes, lower risks of recurrent myocardial infarction and cardiovascular mortality (with some studies even reporting reductions in all-cause mortality) [1,10,11,12,13], and significant improvements in mental health well-being, depression, and anxiety symptoms [14].

Although CRP supports the achievement of therapeutic goals, a significant proportion of patients in real-life clinical practice fail to reach optimal LDL-C targets [15,16,17,18]. This suboptimal LDL-C reduction is frequently attributed to poor adherence to pharmacological therapy and lifestyle recommendations [19,20], which may contribute to patient blame and therapeutic inertia [21]. However, individual variability in response to lipid-lowering therapy (LLT) has been described, influenced by pharmacogenomics factors and additional lipid parameters such as lipoprotein (a) levels [22,23].

The aim of our study is to analyze, in a homogeneous post-MI cohort followed in a CRP, the predictors of lower than expected LDL-C reduction, i.e., a positive residual difference in LDL-C (RD-LDL-C), and specifically to assess the contribution of lifestyle habits to this suboptimal LDL-C reduction.

## 2. Materials and Methods

### 2.1. Population

**Figure 1:**
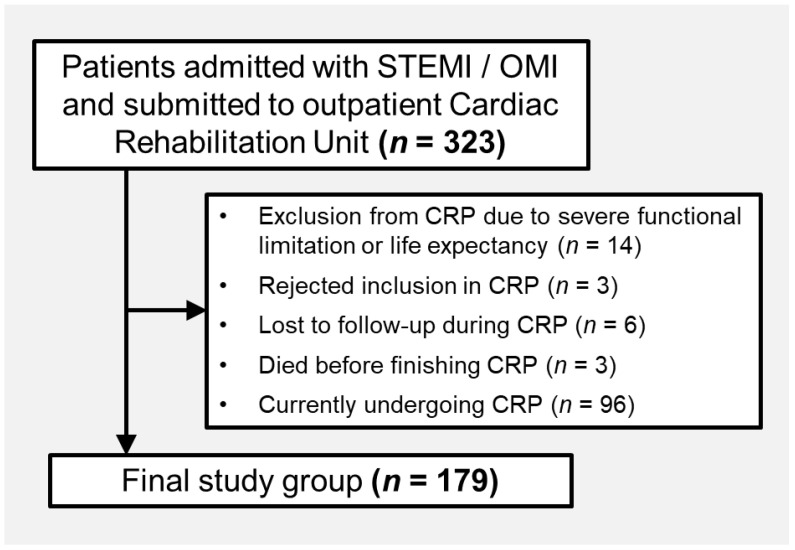
Flowchart of patients included in the study. Abbreviations: CRP = Cardiac Rehabilitation Program. OMI = occlusion myocardial infarction. STEMI = ST-segment elevation acute myocardial infarction.

Our study is based on an ongoing, prospective registry of all ST-segment elevation myocardial infarction (STEMI) or occlusion myocardial infarction (OMI) patients admitted to our high-complexity tertiary care hospital between January 2022 and August 2024 and who were enrolled in the local CRP. After applying several exclusion criteria—including severe functional limitation or limited life expectancy (*n* = 14), refusal to participate in the CRP (*n* = 3), loss to follow-up during the program (*n* = 6), death before completing CRP (*n* = 3), and patients still undergoing CRP at the time of analysis (*n* = 96)—the final study group comprised 179 patients who finished Phase 2 CRP (Figure 1).

We registered baseline clinical characteristics such as age, sex, cardiovascular risk factors, infarct location, Killip class during admission, Global Registry of Acute Coronary Events (GRACE) risk score, echocardiographic left ventricular ejection fraction (LVEF) before discharge, and exercise training modality during CRP.

### 2.2. Cardiac Rehabilitation Program

At our institution, patients with STEMI or OMI are enrolled in the CRP following hospital discharge. Follow-up is carried out in a dedicated outpatient clinic by a multidisciplinary team of cardiologists, physical medicine and rehabilitation specialists, trained nurses, and physiotherapists. Once clinical stabilization is achieved—defined as the absence of chest pain or heart failure decompensation, no hospital readmissions, and normalization of cardiac biomarkers—patients undergo conventional or cardiopulmonary exercise testing (CET/CPET), which assists in stratifying cardiovascular risk as low, intermediate, or high.

Risk stratification following patient inclusion in the CRP is performed according to clinical criteria based on the recommendations of the Spanish Society of Cardiology [24], the European Association of Preventive Cardiology [25], and the American Association of Cardiovascular and Pulmonary Rehabilitation Stratification Algorithm for Risk of Event [26]. Specifically, patients are considered high risk if they present with a LVEF below 40%, Killip class 3 or 4 at admission, complex ventricular arrhythmias, recovered sudden cardiac death, recurrent myocardial infarction or revascularization, functional capacity below 5 metabolic equivalents (METS), hypotensive response to exercise, or evidence of ischemia during low-intensity exercise testing. Based on this assessment, an individualized aerobic and resistance training program is prescribed. This typically consists of at least three months of outpatient sessions, with an additional one to two months of supervised in-hospital training in selected cases.

During this Phase 2 CRP, the cardiology team adjusts pharmacological treatment to optimize cardiovascular risk management, ensure complete smoking cessation, and prescribe cardiac medications when necessary (e.g., antianginal agents or heart failure therapies). At the end of Phase 2, CET/CPET is repeated, updated exercise recommendations are provided for Phase 3, and patients are reassessed in terms of cardiovascular risk control, medication adherence, and improvements in quality of life [14,27,28,29].

### 2.3. Lipid Profile and LLT Analysis

We registered the lipid profile and the prescribed LLT of the cohort before hospital admission and after Phase 2 CRP. For the baseline lipid profile, the electronic clinical history record was revised, and the most recent blood test before admission was registered (median time: 7 [interquartile range: 3, 21] months before admission). The latest blood sample at the end of Phase 2 CRP was also recorded (median time: 6 [interquartile range: 4, 9] months after hospital discharge). The following variables were studied: fasting glucose (mg/dL), glycated hemoglobin (HbA1c, in %), total cholesterol (mg/dL), triglycerides (mg/dL), high-density lipoprotein cholesterol (HDL-C, mg/dL), LDL-C (mg/dL) and non-HDL-C (mg/dL). Additionally, lipoprotein (a) levels were analyzed during admission. LLT at discharge was prescribed by clinical cardiologists during admission according to their clinical judgement.

### 2.4. Corrected Basal LDL-C and Theoretical Potency of LLT

To analyze the theoretical potency for LDL-C reduction of each LLT, data were obtained from several sources as previously published [27]. If no specific data were found regarding an exact combination of drugs, the formula for multiple percentage changes was used [30]:

Final LDL-C with LLT = Initial LDL-C × (1 − % reduction with drug #1) × (1 − % reduction with drug #2) × (1 − % reduction with drug #3) × (1 − % reduction with drug #4)

To calculate the % reduction in LDL-C that a combined treatment would induce, we used the following formula [30]:

% reduction in LDL-C with LLT = [1 − (final LDL-C/initial LDL-C)] × 100

In the subset of patients treated with LLT before admission (*n* = 64, 35.8%), we calculated the corrected basal LDL-C by extrapolating from their measured LDL-C levels before admission and the estimated potency of the LLT which they received at that time. We used the following formula:

$$\mathrm{Corrected}\;\mathrm{basal}\;\mathrm{LDL}\text{-}C=\frac{LDL-C\;with\;LLT}{1-\left(\%\;reduction\;in\;LDL-C\;with\;LLT/100\right)}$$

### 2.5. Residual Difference in LDL-C

RD-LDL-C was defined as the difference between LDL-C measured at the end of Phase 2 CRP and the theoretical LDL-C which was expected to be achieved with the prescribed LLT at that time. For theoretical LDL-C, corrected basal LDL-C levels were used as the reference and the theoretical potency of LLT was established as previously described.

**Figure 2:**
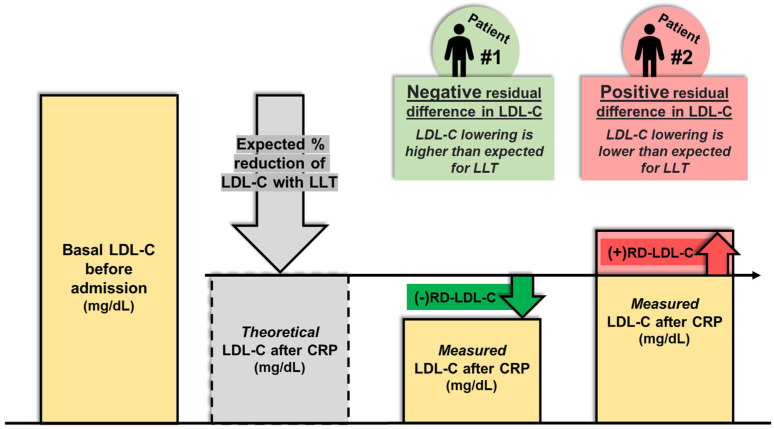
Residual difference in LDL-C after CRP. Abbreviations: CRP = Cardiac Rehabilitation Program. LDL-C = Low-density lipoprotein cholesterol. LLT = Lipid-lowering therapy. (−) RD-LDL-C = Negative residual difference in LDL-C. (+) RD-LDL-C = Positive residual difference in LDL-C.

RD-LDL-C was considered “positive” if >0, indicating lower than expected LDL-C reduction, and “negative” if <0, indicating higher than expected LDL-C reduction (Figure 2).

### 2.6. CRP Outcomes

We analyzed several CRP outcomes at the end of Phase 2. Regarding cardiovascular risk factors, we registered smoking habits, systolic and diastolic blood pressure (in mmHg), weight (in kg), body mass index (BMI), BMI > 30 (% of patients), adherence to the Mediterranean diet (defined as ≥8 points in PREDIMED questionnaire), therapeutic adherence (defined as 4 points in Morisky–Green questionnaire), and the percentage of patients reaching LDL-C < 55 mg/dL and HbA1c < 7%.

Quality of life outcomes were also analyzed, including mean points in the 36-Item Short Form Survey Instrument (SF-36) questionnaire, Patient Health Questionnaire 2-item (PHQ-2, as screening for depression symptoms), and Generalized Anxiety Disorder 2-item (GAD-2, as screening for anxiety symptoms).

Finally, physical fitness variables comprised weekly reported physical activity as METS per week in the International Physical Activity Questionnaire (IPAQ) and peak oxygen consumption (VO_2_) in exercise testing (in mL/kg/min), either estimated on conventional exercise testing or measured on cardiopulmonary exercise testing.

### 2.7. Objetive of the Study

The main objective of our study is to analyze the predictors of lower than expected LDL-C reduction after Phase 2 CRP, i.e., positive RD-LDL-C, and specifically to determine whether lifestyle habits can significantly contribute to this suboptimal LDL-C reduction.

### 2.8. Ethics

The study was conducted in accordance with the ethical guidelines of the World Medical Association Declaration of Helsinki and approved by the Ethics Committee for Drug Research (CEIm) of Hospital Clinico Universitario de Valencia (protocol code: 2019/262 and date of approval: 26 May 2020). Informed consent was obtained from all subjects involved in the study.

### 2.9. Statistical Analysis

A one-sample Kolmogorov-Smirnov test was used to test normal data distribution. For continuous parametric variables, data are expressed as mean ± standard deviation and analyzed by Student’s *t* test. Continuous non-parametric variables are shown as median plus interquartile range and compared with a Mann–Whitney U test. Qualitative variables are presented as percentages and compared by chi-square test or Fisher’s exact test.

Univariate analyses were performed to identify variables associated with positive RD-LDL-C after Phase 2 CRP. Variables with *p*-values < 0.1 in univariate analysis were included as cofactors in a multivariate binary logistic regression model. A stepwise approach was adopted to avoid overfitting. Clinical variables during admission were included in the first step. Lipid and metabolic profile variables before admission were added in the second step. Nagelkerke’s R^2^ and Chi-square values were computed to test the model’s fit. Multicollinearity between variables was ruled out by means of variance inflation factor (normal if <5) and tolerance statistic (normal if >0.2). The predicted probability in the final binary logistic regression model was used to predict positive RD-LDL-C after Phase 2 CRP. Receiver operating characteristic (ROC) curves were computed to independently analyze the discrimination ability of the model. An area under the ROC curve (AUC) > 0.8 was considered excellent.

Statistical significance was considered for 2-tailed *p*-values < 0.05. The SPSS statistical package version 26.0 (IBM Corp., Armonk, NY, USA) was used.

## 3. Results

### 3.1. Cohort Description

Our cohort comprised 179 patients who completed Phase 2 CRP (Table 1). The mean age was 63.04 ± 10.56 years, most were male (*n* = 152, 84.9%), and hypercholesterolemia was the most prevalent cardiovascular risk factor (*n* = 160, 89.4%). The mean GRACE risk score was 118.3 ± 28.88 points and anterior and inferior STEMI were the most prevalent infarct locations (*n* = 80, 44.7% and *n* = 78, 43.6%, respectively). The mean LVEF was 52.29 ± 10.57% and 35.2% of patients had an LVEF < 50%. Ambulatory training was provided to 142 (79.3%) patients and 37 (20.7%) patients received additional supervised in-hospital training sessions.

Regarding lipid profile prior to admission, total cholesterol was 199.83 ± 54.72 mg/dL, median triglycerides (TG) was 124 [90, 169] mg/dL, mean HDL was 46.25 ± 11.1 mg/dL, mean LDL-C was 131.66 ± 45.02 mg/dL, and mean corrected basal LDL-C was 159.72 ± 45.14 mg/dL. A substantial reduction in LDL-C was noted after Phase 2 CRP - mean LDL-C was 43.05 ± 13.8 mg/dL, and 153 (85.5%) patients achieved target LDL-C < 55 mg/dL. Median lipoprotein (a) levels were 28 [12.25, 76.75] mg/dL.

After Phase 2 CRP, most previous smokers achieved smoking cessation (86.2%), 88.1% of patients were adherent to the Mediterranean diet as assessed by the PREDIMED questionnaire, 89.8% had perfect adherence to pharmacological therapy on the Morisky–Green questionnaire, and patients were highly active on the IPAQ questionnaire, performing a median physical activity of 3545 [1980, 6132] METS per week. Mean increase in peak VO_2_ was 3.6 ± 4.73 mL/kg/min.

### 3.2. Lipid-Lowering Therapy Before and After Phase 2 CRP

LLT before and after Phase 2 CRP is presented in Table 2. At hospital discharge, most patients (89.4%) received a high-intensity statin (atorvastatin 40–80 mg o.d. or rosuvastatin 20–30 mg o.d.). Combination therapy with ezetimibe 10 mg o.d. was prescribed in 97 (54.2%) patients, although the use of bempedoic acid or PCSK9 inhibitors was rare (both 0.6%). The estimated theoretical potency of LLT at discharge was 58.99 ± 10.96 %.

After Phase 2 CRP, most patients (92.2%) were treated with a high-intensity statin in combination with ezetimibe 10 mg o.d. (91.6%). Additionally, an increased use of bempedoic acid 180 mg o.d. (*n* = 19, 10.6%), PCSK9 inhibitors (*n* = 24, 13.4%), and inclisiran (*n* = 6, 3.4%) was observed. A significant increase in the theoretical potency of LLT was evidenced after Phase 2 CRP (67.44 ± 7.51%; the median change was 6 [0, 15] %, *p* < 0.001).

### 3.3. Residual Difference in LDL-C

After Phase 2 CRP, 54 (30.2%) patients exhibited positive RD-LDL-C, indicating a lower than expected LDL-C reduction (Table 1). These patients were older (65.84 ± 11.77 vs. 61.83 ± 9.8 years, *p* = 0.03), more frequently male (98.1% vs. 79.2%, *p* = 0.001), had a higher prevalence of hypertension (68.5% vs. 52%, *p* = 0.04) but a lower prevalence of diabetes mellitus (11.1% vs. 28.8%, *p* = 0.01), showed higher GRACE risk scores (126.61 ± 33.08 vs. 114.72 ± 26.2 points, *p* = 0.02), and less frequently participated in supervised in-hospital training (9.3% vs. 20.7%, *p* = 0.02).

Regarding lipid profile before admission, patients with positive RD-LDL-C had lower total cholesterol (186.04 ± 54.05 vs. 205.78 ± 54.13 mg/dL, *p* = 0.03), TG (117.54 [84, 150.25] vs. 156.76 [97, 184] mg/dL, *p* = 0.002), LDL-C (121.46 ± 42.91 vs. 136.06 ± 45.36 mg/dL, *p* = 0.04) and corrected basal LDL-C (143.09 ± 42.03 vs. 166.9 ± 44.69 mg/dL, *p* = 0.001). However, higher levels of lipoprotein (a) were observed (49 [15, 90] vs. 25 [12, 60] mg/dL, *p* = 0.03).

This subgroup showed higher LDL-C levels after Phase 2 CRP (51.72 ± 10.13 vs. 39.3 ± 13.52 mg/dL, *p* < 0.001) and were less likely to reach target LDL-C < 55 mg/dL (66.7% vs. 93.6%, *p* < 0.001). However, these patients received more potent LLT after CRP (theoretical potency: 69.81 ± 7.07 vs. 66.41 ± 7.48%, *p* = 0.005) and underwent more intensive uptitration of LLT during Phase 2 (increase in theoretical potency: 9 [3.75, 16.25] vs. 0 [0, 13] %, *p* < 0.001).

No differences were found with respect to adherence to the Mediterranean diet (92.5% vs. 86.2%, *p* = 0.24), therapeutic adherence (92.5% vs. 88.6%, *p* = 0.44) or weekly physical activity (4531 [1998, 6774] vs. 3339 [1980, 5670] METS per week, *p* = 0.1), although patients with positive RD-LDL-C tended to have numerically better indicators on these parameters. Similarly, the improvement in peak VO_2_ was comparable between this subset and patients with negative RD-LDL-C (3.49 ± 4.67 vs. 3.64 ± 4.77 mL/kg/min, *p* = 0.84).

### 3.4. Predictors of Positive Residual Difference in LDL-C

**Figure 3:**
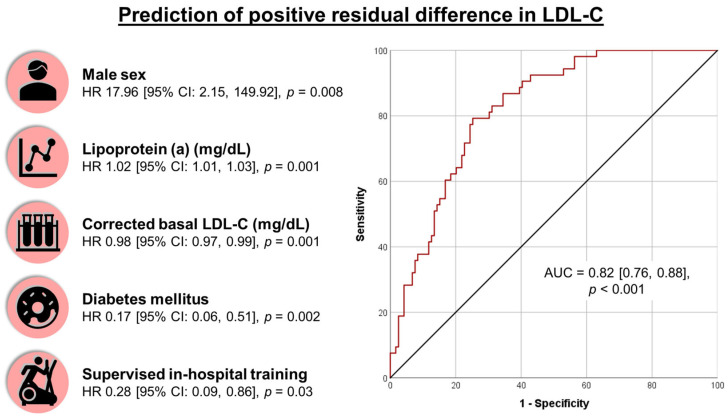
Prediction of positive residual difference in LDL-C after CRP. Predictors of positive residual difference in LDL-C (**left**) and ROC curve analysis (**right**). Abbreviations: AUC = Area under the curve. CI = Confidence interval. HR = Hazard ratio. LDL-C = Low-density lipoprotein cholesterol. ROC = Receiver operating characteristic.

We performed a multivariable binary logistic regression analysis to identify predictors of positive RD-LDL-C in our cohort. The final model (Table 3, Figure 3) was statistically significant (Chi-squared = 53.06, *p* < 0.001, Nagelkerke’s R^2^ = 0.376), correctly classified 69% of cases, and showed an excellent discrimination ability (AUC = 0.82 [0.76, 0.88], *p* < 0.001).

Two variables associated with an increased likelihood of positive RD-LDL-C: male sex (HR 17.96 [2.15, 149.92], *p* = 0.008) and higher lipoprotein (a) levels (HR 1.02 [1.01, 1.03] per mg/dL, *p* = 0.001). Three variables associated with a reduced likelihood of positive RD-LDL-C: diabetes mellitus (HR 0.17 [0.06, 0.51], *p* = 0.002), supervised in-hospital training during Phase 2 CRP (HR 0.28 [0.09, 0.86], *p* = 0.03), and higher levels of corrected basal LDL-C (HR 0.98 [0.97, 0.99] per mg/dL, *p* = 0.001). Supervised in-hospital training exhibited the most modest predictive value.

## 4. Discussion

In our study, we examined the predictors of lower than expected LDL-C reduction in a closely monitored cohort of post-MI patients undergoing Phase 2 CRP. Nearly one-third of the cohort exhibited positive RD-LDL-C. In this subgroup, patients underwent uptitration of LLT, and they received more potent LLT by the end of Phase 2 CRP. Male sex and higher lipoprotein (a) levels were associated with positive RD-LDL-C, whereas diabetes mellitus, higher corrected basal LDL-C levels, and supervised in-hospital training during CRP were linked to a reduced likelihood of positive RD-LDL-C. No association was found with adherence to the Mediterranean diet, reported weekly physical activity, or change in peak VO_2_.

### 4.1. Post-MI LDL-C Goals and Cardiac Rehabilitation

Current guidelines indicate that all MI patients should reach and maintain a target LDL-C below 55 mg/dL, along with a reduction of at least 50% from basal LDL-C levels [1]. This recommendation is based on the causal role of LDL-C in the atherosclerotic process that underlies most cases of MI [2] and is supported by evidence demonstrating that greater LDL-C reductions are associated with more pronounced decreases in cardiovascular risk, regardless of the therapeutic strategy employed [31]. The beneficial impact of lowering LDL-C depends both on the patient’s cardiovascular risk and the extent of cholesterol reduction. Therefore, even a modest decrease in LDL-C levels may confer significant benefits in individuals at high or very high cardiovascular risk [32].

CRP can play a key role in achieving therapeutic LDL-C targets through exercise training, lifestyle interventions, promotion of therapeutic adherence, and optimization of LLT in cases with out-of-range LDL-C levels. For instance, in our cohort of patients monitored during Phase 2 CRP, over 85% reached the optimal LDL-C target. Our intervention resulted in very high adherence to pharmacological therapy and the Mediterranean diet, and patients were highly active after Phase 2 CRP. Nevertheless, it should be noted that substantial uptitration of LLT was also performed in patients with suboptimal LDL-C levels, which undoubtedly contributed to this excellent LDL-C control. However, in our clinical setting, reimbursement criteria by the local healthcare system restrict the usage of parenteral LLT such as PCSK9i or inclisiran to a limited subset of uncontrolled patients.

### 4.2. Lifestyle Habits, Therapeutic Adherence, and Therapeutic Inertia

In contrast, a considerable proportion of patients fail to achieve optimal LDL-C goals in routine clinical practice [15,16,17,18]. Challenges in reaching lipid therapeutic targets are often attributed to poor treatment adherence, fear of side effects, and therapeutic inertia. The latter refers to the failure to initiate or intensify LLT despite not reaching LDL-C goals, especially when the patient is close to achieving them. Therapeutic inertia is a well-recognized barrier to improving clinical care and health outcomes [21,33,34,35].

In routine clinical practice, it is frequently assumed that lifestyle improvements—such as dietary changes or increased physical activity—will suffice to achieve target LDL-C levels, based on the premise that the patient has not yet effectively implemented these modifications. Moreover, it is common to postpone a more intensive medical intervention until a second visit, once laboratory analyses confirm that a therapeutic adjustment is indeed necessary. This delay in optimal cholesterol control may, in turn, increase the risk of cardiovascular events [19,20].

While lifestyle habits remain the cornerstone of secondary prevention, the response to LLT can vary significantly among individuals. This interindividual variability is influenced by multiple factors, including the presence of genetic polymorphisms, specific clinical characteristics, and the impact of other lipid abnormalities such as elevated triglycerides or lipoprotein (a) levels, among others [23,36,37,38]. As a result, even when patients strictly adhere to medical and lifestyle recommendations, a suboptimal or lower than expected therapeutic response may still be observed.

In our study, we observed that patients who do not achieve the expected reduction in LDL-C do not exhibit poorer treatment adherence: 90% demonstrate perfect therapeutic adherence. Moreover, approximately 90% follow a Mediterranean diet, and no differences were detected in physical activity levels according to the IPAQ questionnaire. We also found no indirect evidence suggesting that patients were overreporting their level of physical activity, as this subgroup showed a similar improvement in functional capacity (VO_2_) as patients with negative RD-LDL-C. These exceptionally high adherence rates, although desirable, may have positively influenced the overall effectiveness and observed outcomes of our CRP. In our view, these findings should encourage a more proactive approach to pharmacological management, avoiding the attribution of unmet targets solely to a lack of adherence to lifestyle habits. Instead, it is essential to acknowledge that interindividual variability exists, which may require a more intensive approach in certain cases.

### 4.3. Predictors of Positive RD-LDL-C

According to our results, male patients were more likely to achieve a lower than expected LDL-C reduction. Traditionally, female patients have been observed to have poorer secondary prevention control, including LDL-C goals, even when CRP follow-up has been provided [39]. This discrepancy could be explained by several factors. First, women have lower enrollment rates in CRP than men and experience higher dropout rates, mainly due to advanced age, comorbidities, and family responsibilities [40,41]. Moreover, women are less likely to receive intensive LLT [42,43] and exhibit lower adherence to LLT [44,45]. Our study suggests that if proactive intensification of LLT is performed and therapeutic adherence is achieved in post-MI women followed in a CRP, the observed relative LDL-C reductions can exceed those of their male counterparts.

Lipoprotein (a) is gaining increasing relevance in clinical practice since it has been shown to increase cardiovascular risk irrespective of LDL-C levels. In our study, lipoprotein (a) levels were significantly and independently associated with a positive RD-LDL-C. The use of the Friedewald formula, applied to most patients in our study, may overestimate LDL-C levels in the presence of elevated lipoprotein (a) concentrations due to the overlap in particle densities. This phenomenon has been previously reported in the literature [46,47,48]. Nevertheless, it remains unclear whether adjusting LDL-C values based on lipoprotein (a) levels should be implemented to improve cardiovascular risk assessment [49].

On the other hand, we observed that patients with diabetes mellitus showed higher than expected LDL-C reduction. Diabetes is associated with a specific dyslipidemic profile, characterized by higher TG levels, low HDL-C, high apolipoprotein-B levels, and smaller, denser LDL-C particles [50]. One possible explanation for the greater reduction in LDL-C in these patients could be the influence of antidiabetic medications, which may impact cholesterol levels in this context. In fact, SGLT-2 inhibitors have been shown to benefit patients with acute myocardial infarction and improve metabolic levels through pleiotropic effects [51]. Another possible explanation is the metabolic and physiological differences between diabetic and non-diabetic patients, which may affect the response to LLT. One study found that both diabetic and non-diabetic patients demonstrated similar efficacy in LDL-C reduction with LLT [52]. However, other studies have observed that diabetic patients seem to respond better to LLT during CRP [53], even showing a greater reduction in the risk of major coronary events (42%) compared to non-diabetic patients (32%) [54].

Another variable associated with higher than expected LDL-C reduction was higher baseline LDL-C levels. This suggests that the higher the baseline LDL-C, the greater the relative effect of the LLT. This phenomenon might be partially explained by regression to the mean, similar to what has been described for HDL-C improvement following statin therapy relative to baseline HDL-C levels [28,55]. Regression to the mean is a statistical phenomenon whereby extreme values of a quantitative variable, such as LDL-C, tend to move closer to the average upon subsequent measurements. This shift does not necessarily reflect the true effect of an intervention but may simply result from natural variability. In studies like ours, which include measurements before and after treatment, this effect can at least partially influence LDL-C levels. Additionally, in our study, baseline LDL-C levels were estimated using formulas in patients who were already on LLT, which introduces extra variability and could further contribute to this bias. Another potential explanation for a greater reduction in LDL-C among patients with higher baseline LDL-C levels is that patients with a positive RD-LDL-C may have had less healthy lifestyle habits prior to MI. However, we do not have data to support this hypothesis, as IPAQ and PREDIMED questionnaire scores were similar between the two subgroups.

Finally, patients who underwent supervised in-hospital training during CRP also showed higher than expected LDL-C reduction. Generally, home-based CRP have been shown to have similar efficacy in optimizing lipid profiles compared to center-based CRP [56,57]. In our cohort, however, participation in supervised in-hospital training appeared to confer additional benefits regarding LDL-C lowering, supporting the notion that more intensive interventions during a CRP can provide further LDL-C reductions [11] and improve prognosis [58].

### 4.4. Implications for Clinical Practice

Adherence to healthy lifestyle recommendations and pharmacological treatment is crucial in clinical practice in post-MI patients, as it can significantly influence the achievement of secondary prevention goals and improve long-term prognosis. Our study suggests that although lifestyle measures (e.g., supervised in-hospital training) may positively impact LDL-C reduction, in closely monitored post-MI patients followed in a CRP, most of the suboptimal response to LLT can be attributed to factors such as male sex, higher lipoprotein (a) levels, lower baseline LDL-C levels, and the absence of diabetes mellitus. Therefore, clinicians should be aware that a failure to achieve target LDL-C levels should not be solely attributed to inadequate adherence to lifestyle recommendations or pharmacological therapy. Instead, a proactive approach to uptitrating LLT while considering individual variability in treatment response is warranted to enable earlier goal attainment and avoid patient blame.

### 4.5. Limitations

Our study has several limitations that warrant discussion. Firstly, it is an observational study without a comparison group, which makes it more susceptible to potential biases. Additionally, it is a single-center study conducted at our local CRP, with patient selection criteria tailored to the specific characteristics of our center. This may limit the generalizability of our results to other CRP or patients treated in different outpatient settings. The unusually high adherence rates observed in our CRP, although desirable, may have influenced the overall effectiveness of the program and the observed outcomes, therefore limiting the generalizability of our findings to broader, less-motivated populations. Another important limitation is the absence of a validation cohort, which restricts the external validity of our results. Furthermore, for patients already on LLT before admission, the method used to estimate corrected basal LDL-C introduces variability within the study population, which should be recognized as a potential limitation. Consequently, our study should be interpreted with caution and considered primarily as hypothesis-generating.

## 5. Conclusions

More than one-third of post-MI patients demonstrated lower than expected LDL-C reduction (positive RD-LDL-C) following CRP, a finding that could not be attributed to poor adherence to pharmacological therapy or lifestyle recommendations. These findings suggest that a personalized approach to prescribing and uptitrating LLT may help achieve LDL-C targets, particularly in MI patients with healthy lifestyle habits who exhibit a lower response to LLT.

## Figures and Tables

**Table 1:** Clinical variables, cardiovascular risk factors, quality of life outcomes, and physical fitness variables according to residual difference in LDL-C.

Variable	All Patients (*n* = 179)	Negative RD-LDL-C (*n* = 125)	Positive RD-LDL-C (*n* = 54)	*p*-Value
**Clinical variables**				
Age (years)	63.04 ± 10.56	61.83 ± 9.8	65.84 ± 11.77	0.03
Male sex (%)	152 (84.9)	99 (79.2)	53 (98.1)	0.001
Hypercholesterolemia (%)	160 (89.4)	115 (92)	45 (83.3)	0.08
Hypertension (%)	102 (57)	65 (52)	37 (68.5)	0.04
Diabetes mellitus (%)	42 (23.5)	36 (28.8)	6 (11.1)	0.01
Killip class ≥ 2 (%)	51 (28.5)	31 (24.8)	20 (37)	0.1
GRACE risk score	118.3 ± 28.88	114.72 ± 26.2	126.61 ± 33.08	0.02
Infarct location				0.46
Anterior (%)	80 (44.7)	51 (40.8)	29 (53.7)	
Inferior (%)	78 (43.6)	58 (46.4)	20 (37)	
Lateral (%)	12 (6.7)	9 (7.2)	3 (5.6)	
OMI (%)	9 (5)	7 (5.6)	2 (3.7)	
LVEF (%)	52.29 ± 10.57	52.74 ± 10.7	51.26 ± 10.28	0.39
LVEF < 50% (%)	63 (35.2)	44 (35.2)	19 (35.2)	1
Exercise training modality during CRP				0.02
Ambulatory training (%)	142 (79.3)	93 (74.4)	49 (90.7)	
Supervised in-hospital training (%)	37 (20.7)	32 (25.6)	5 (9.3)	
**Lipid and metabolic profile before admission**				
Fasting blood glucose (mg/dL)	99.59 ± 23.69	103.27 ± 24.66	91.15 ± 18.95	0.002
Total cholesterol (mg/dL)	199.83 ± 54.72	205.78 ± 54.13	186.04 ± 54.05	0.03
Triglycerides (mg/dL)	124 [90, 169]	156.76 [97, 184]	117.54 [84, 150.25]	0.002
HDL-C (mg/dL)	46.25 ± 11.1	45.99 ± 9.99	46.85 ± 13.41	0.67
Non-HDL-C (mg/dL)	153.58 ± 51.4	159.79 ± 51.92	139.19 ± 47.58	0.01
LDL-C (mg/dL)	131.66 ± 45.02	136.06 ± 45.36	121.46 ± 42.91	0.04
Corrected basal LDL-C (mg/dL) ^#^	159.72 ± 45.14	166.9 ± 44.69	143.09 ± 42.03	0.001
LLT before admission (%)	64 (35.8)	47 (37.6)	17 (31.5)	0.43
HbA1c (%) *	6.25 ± 1.51	6.5 ± 1.33	5.32 ± 1.81	0.01
**Lipid and metabolic profile after CRP**				
Fasting blood glucose (mg/dL)	94.91 ± 18.7	97.33 ± 20.08	89.41 ± 13.72	0.009
Median change (mg/dL)	−4.5 [−12.8, 5.75]	−5.5 [−13.25, 5.5]	−3 [−12, 6]	0.47
Total cholesterol (mg/dL)	102.63 ± 17.85	99.44 ± 17.81	109.96 ± 15.79	<0.001
Mean change (mg/dL)	−97.31 ± 52.44	−106.56 ± 50.23	−76.07 ± 51.68	<0.001
Triglycerides (mg/dL)	86.5 [65–118]	93 [68.25, 122.5]	78 [59.5, 103]	0.02
Median change (mg/dL)	−36.5 [−73.25, −6.75]	−40 [−75.5, −12.5]	−27 [−67.25, 0]	0.1
HDL-C (mg/dL)	43.28 ± 9.03	42.76 ± 8.71	44.48 ± 9.71	0.24
Median change (mg/dL)	−3 [−8, 2]	−3 [−8, 2]	−3 [−7.25, 2.5]	0.99
Non-HDL-C (mg/dL)	59.35 ± 16.17	56.69 ± 16.64	65.48 ± 13.24	0.001
Mean change (mg/dL)	−94.26 ± 49.72	−103.22 ± 48.24	−73.7 ± 47.3	<0.001
LDL-C (mg/dL)	43.05 ± 13.8	39.3 ± 13.52	51.72 ± 10.13	<0.001
Mean change (mg/dL)	−88.61 ± 42.71	−96.76 ± 41.13	−69.74 ± 40.62	<0.001
Theoretical LDL-C after CRP (mg/dL)	50.82 ± 16.18	54.79 ± 16.24	41.64 ± 11.8	<0.001
RD-LDL-C (mg/dL)	−7.54 [−16.8, 2.8]	−13.6 [−20.12, −6.91]	8.92 [3.97, 14.39]	<0.001
LDL-C < 55 mg/dL after CRP (%)	153 (85.5)	117 (93.6)	36 (66.7)	<0.001
HbA1c (%)	6 ± 0.6	6.06 ± 0.66	5.86 ± 0.39	0.04
Median change (%)	0.1 [−0.3, 0.4]	0 [−0.4, 0.28]	0.3 [0.2, 0.4]	0.13
HbA1c < 7% after CRP (%)	168 (94.9)	116 (93.5)	52 (98.1)	0.21
**Other cardiovascular risk factors**				
Lipoprotein (a) (mg/dL)	28 [12.25, 76.75]	25 [12, 60]	49 [15, 90]	0.03
Smoking habit before CRP (%)	87 (48.6)	61 (48.8)	26 (48.1)	0.94
Smoking habit after CRP (%)	12 (6.7)	6 (4.8)	6 (11.1)	0.12
Systolic pressure before CRP (mmHg)	124.6 ± 15.42	125 ± 15.52	123.67 ± 15.28	0.6
Systolic pressure after CRP (mmHg)	115.14 ± 9.69	115.02 ± 9.44	115.43 ± 10.35	0.8
Median change (mmHg)	−10 [−20, 0]	−10 [−20, −4]	−6 [−16, 0]	0.2
Diastolic pressure before CRP (mmHg)	77.6 ± 8.51	78.22 ± 7.78	76.15 ± 9.1	0.71
Diastolic pressure after CRP (mmHg)	73.54 ± 7.12	74.36 ± 6.87	72.95 ± 7.56	0.82
Median change (mmHg)	−4 [−7, 0]	−4 [−8, −2]	−4 [−6, 0]	0.87
Weight before CRP (kg)	79.85 ± 13.4	79.93 ± 13.99	79.68 ± 12.06	0.91
Weight after CRP (kg)	78.03 ± 12.78	78.18 ±13.14	77.68 ± 12	0.81
Median change (kg)	−1 [−4, 1.5]	−1.5 [−4, 1.5]	−0.7 [−4.63, 1.25]	0.81
BMI before CRP	27.47 ± 4.2	27.8 ± 4.48	26.7 ± 3.41	0.11
BMI after CRP	26.87 ± 3.98	27.22 ± 4.18	26.08 ± 3.39	0.08
Median change	−0.33 [−1.49, 0.37]	−0.41 [−1.48, 0.47]	−0.24 [−1.53, 0.21]	0.92
BMI ≥ 30 before CRP (%)	39 (21.8)	31 (24.8)	8 (14.8)	0.14
BMI ≥ 30 after CRP (%)	34 (19)	27 (21.6)	7 (13)	0.18
Adherence to Mediterranean diet after CRP (≥8 points in PREDIMED)	155 (88.1)	106 (86.2)	49 (92.5)	0.24
Therapeutic adherence after CRP (4 points in Morisky–Green)	158 (89.8)	109 (88.6)	49 (92.5)	0.44
**Quality of life outcomes**				
SF-36 (mean) before CRP (points)	64 ± 19.4	64.19 ± 18.7	63.54 ± 21.09	0.84
SF-36 (mean) after CRP (points)	71.27 ± 18.88	71.01 ± 19.04	71.88 ± 18.66	0.78
Median change (points)	5.61 [−3.66, 18.38]	5.28 [−3.73, 15.48]	5.88 [−3.08, 21.48]	0.49
PHQ-2 before CRP (points)	1 [0, 2]	1 [0, 2]	1 [0, 2]	0.74
PHQ-2 after CRP (points)	0 [0, 2]	0 [0, 2]	0 [0, 2]	0.43
Median change (points)	0 [−1, 0]	0 [−1, 0]	0 [−1, 0]	0.22
GAD-2 before CRP (points)	2 [0, 3]	2 [0, 3]	2 [0, 3]	0.74
GAD-2 after CRP (points)	1 [0, 2]	1 [0, 2]	1 [0, 2]	0.94
Median change (points)	0 [−2, 0]	0 [−1, 0]	0 [−2, 0]	0.99
**Physical fitness variables**				
IPAQ before CRP (METS/week)	1386 [641.25, 2772]	1386 [495, 2772]	1386 [693, 2826.75]	0.5
IPAQ after CRP (METS/week)	3545 [1980, 6132]	3339 [1980, 5670]	4531 [1998, 6774]	0.1
Median change (METS/week)	1636 [461.75, 3572.63]	1565 [489, 3031.5]	2528.75 [373.88, 5311.5]	0.19
Peak VO_2_ before CRP (mL/kg/min)	25.66 ± 9.26	26.39 ± 9.47	23.96 ± 8.6	0.11
Peak VO_2_ after CRP (mL/kg/min)	29.25 ± 10.43	30.03 ± 10.6	27.44 ± 9.89	0.13
Mean change (mL/kg/min)	3.6 ± 4.73	3.64 ± 4.77	3.49 ± 4.67	0.84

^#^ Corrected basal LDL-C is defined as the LDL-C before admission (in patients without previous LLT) or the corrected LDL-C (in patients with previous LLT). Readers are referred to Section 2.4 for further details. * HbA1c measurement was unavailable in 122 (68.2%) patients before admission. Abbreviations: BMI = Body mass index. CRP = Cardiac Rehabilitation Program. GAD-2 = Generalized Anxiety Disorder 2-item. GRACE = Global Registry of Acute Coronary Events. HbA1c = Glycated hemoglobin. IPAQ = International Physical Activity Questionnaire. LDL-C = Low-density lipoprotein cholesterol. LVEF = Left ventricular ejection fraction. METS = Metabolic equivalents. OMI = Occlusion myocardial infarction. PHQ-2 = Patient Health Questionnaire 2-item. PREDIMED = Prevención con Dieta Mediterránea. RD-LDL-C = Residual difference in low-density lipoprotein cholesterol. SF-36 = 36-Item Short Form Survey Instrument. VO_2_ = Oxygen consumption.

**Table 2:** LLT at discharge and after Phase CRP according to residual difference in LDL-C.

	All Patients (*n* = 179)	Negative RD-LDL-C (*n* = 125)	Positive RD-LDL-C (*n* = 54)	*p*-Value
**LLT at discharge**				
Statins				0.74
No statin	6 (3.4)	4 (3.2)	2 (3.7)	
Fluvastatin 80 mg o.d.	1 (0.6)	1 (0.8)	0 (0)	
Pitavastatin 4 mg o.d.	1 (0.6)	1 (0.8)	0 (0)	
Atorvastatin 20 mg o.d.	2 (1.1)	2 (1.6)	0 (0)	
Atorvastatin 40 mg o.d.	20 (11.2)	16 (12.8)	4 (7.4)	
Atorvastatin 60 mg o.d.	3 (1.7)	2 (1.6)	1 (1.9)	
Atorvastatin 80 mg o.d.	63 (35.2)	39 (31.2)	24 (44.4)	
Rosuvastatin 10 mg o.d.	9 (5)	7 (5.6)	2 (3.7)	
Rosuvastatin 15 mg o.d.	2 (1.1)	1 (0.8)	1 (1.9)	
Rosuvastatin 20 mg o.d.	71 (39.7)	51 (40.8)	20 (37)	
Rosuvastatin 30 mg o.d.	1 (0.6)	1 (0.8)	0 (0)	
Ezetimibe 10 mg o.d.	97 (54.2)	72 (57.6)	25 (46.3)	0.16
Bempedoic acid 180 mg o.d.	1 (0.6)	1 (0.8)	0 (0)	0.51
PCSK9 inhibitors	1 (0.6)	1 (0.8)	0 (0)	0.51
Inclisiran	0 (0)	0 (0)	0 (0)	-
Fibrates	0 (0)	0 (0)	0 (0)	-
Theoretical potency of LLT at discharge (% reduction of LDL-C)	58.99 ± 10.96	59.41 ± 11.03	58.04 ± 10.83	0.44
**LLT after CRP**				
Statins				0.04
No statin	1 (0.6)	1 (0.8)	0 (0)	
Pitavastatin 1 mg o.d.	1 (0.6)	0 (0)	1 (1.9)	
Pitavastatin 4 mg o.d.	2 (1.1)	2 (1.6)	0 (0)	
Atorvastatin 20 mg o.d.	1 (0.6)	0 (0)	1 (1.9)	
Atorvastatin 40 mg o.d.	23 (12.8)	18 (14.4)	5 (9.3)	
Atorvastatin 80 mg o.d.	45 (25.1)	27 (21.6)	18 (33.3)	
Rosuvastatin 10 mg o.d.	9 (5)	8 (6.4)	1 (1.9)	
Rosuvastatin 20 mg o.d.	85 (47.5)	65 (52)	20 (37)	
Rosuvastatin 30 mg o.d.	5 (2.8)	2 (1.6)	3 (5.6)	
Rosuvastatin 40 mg o.d.	7 (3.9)	2 (1.6)	5 (9.3)	
Ezetimibe 10 mg o.d.	164 (91.6)	111 (88.8)	53 (98.1)	0.04
Bempedoic acid 180 mg o.d.	19 (10.6)	8 (6.4)	11 (20.4)	0.005
PCSK9 inhibitors	24 (13.4)	15 (12)	9 (16.7)	0.5
Inclisiran	6 (3.4)	5 (4)	1 (1.9)	0.52
Fibrates	1 (0.6)	1 (0.8)	0 (0)	0.54
Theoretical potency of LLT after CRP (% reduction of LDL-C)	67.44 ± 7.51	66.41 ± 7.48	69.81 ± 7.07	0.005
Median change (%)	6 [0, 15]	0 [0, 13]	9 [3.75, 16.25]	<0.001

Abbreviations: CRP = Cardiac Rehabilitation Program. LDL-C = Low-density lipoprotein cholesterol. LLT = Lipid-lowering therapy. PCSK9 = Proprotein convertase subtilisin/kexin type 9.

**Table 3:** Predictors of positive residual difference in LDL-C on multivariable logistic regression analysis.

Variable	HR [95% CI]	Change in Model’s Nagelkerke’s R^2^	Change in Model’s Chi-Squared	*p*-Value
**Model 1** **—clinical variables**				
Age (years)	1.02 [0.97, 1.08]	-	-	0.4
Male sex (%)	16.02 [2.08, 123.34]	0.107	14.07	0.008
Hypercholesterolemia (%)	0.4 [0.13, 1.27]	-	-	0.12
Hypertension (%)	1.87 [0.86, 4.07]	-	-	0.11
Diabetes mellitus (%)	0.26 [0.1, 0.69]	0.056	7.87	0.006
GRACE risk score	1.01 [0.99, 1.03]	-	-	0.43
Supervised in-hospital training	0.26 [0.09, 0.73]	0.055	7.96	0.01
**Model 2** **—clinical variables and lipid and metabolic profile variables before admission**				
Male sex	17.96 [2.15, 149.92]	0.117	14.79	0.008
Diabetes mellitus	0.17 [0.06, 0.51]	0.07	10.85	0.002
Supervised in-hospital training	0.28 [0.09, 0.86]	0.035	5.69	0.03
Fasting blood glucose (mg/dL)	1 [0.98, 1.02]	-	-	0.91
Total cholesterol (mg/dL)	0.99 [0.94, 1.05]	-	-	0.82
Triglycerides (mg/dL)	0.99 [0.97, 1.01]	-	-	0.22
Non-HDL-C (mg/dL)	1.04 [0.94, 1.15]	-	-	0.43
LDL-C (mg/dL)	0.98 [0.89, 1.07]	-	-	0.61
Corrected basal LDL-C (mg/dL)	0.98 [0.97, 0.99]	0.077	11.2	0.001
HbA1c (%)	0.58 [0.17, 1.92]	-	-	0.37
Lipoprotein (a) (mg/dL)	1.02 [1.01, 1.03]	0.077	10.53	0.001

Abbreviations: CI = Confidence interval. HR = Hazard ratio. LDL-C = Low-density lipoprotein cholesterol.

## Data Availability

The data presented in this study are available on request from the corresponding authors. The data are not publicly available due to ethical restrictions.

## Abbreviations

The following abbreviations are used in this manuscript:

AUC	Area under the curve
BMI	Body mass index
CET	Conventional exercise testing
CI	Confidence Interval
CPET	Cardiopulmonary exercise testing
CRP	Cardiac Rehabilitation Program
GAD-2	Generalized Anxiety Disorder 2-item
GRACE	Global Registry of Acute Coronary Events
HDL-C	High-density lipoprotein cholesterol
HR	Hazard ratio
IPAQ	International Physical Activity Questionnaire
LDL-C	Low-Density Lipoprotein Cholesterol
LLT	Lipid-lowering therapy
LVEF	Left Ventricular Ejection Fraction
METS	Metabolic equivalents
MI	Myocardial Infarction
OMI	Occlusion Myocardial Infarction
PHQ-2	Patient Health Questionnaire 2-item
RD-LDL-C	Residual difference in LDL-C
ROC	Receiver operating characteristic
SF-36	36-Item Short Form Survey Instrument
SPSS	Statistical Package for the Social Sciences
STEMI	ST-segment Elevation Myocardial Infarction
TG	Triglycerides
VO_2_	Oxygen consumption
